# Laser Doppler blood flowmeter as a useful instrument for the early detection of lower extremity peripheral arterial disease in hemodialysis patients: an observational study

**DOI:** 10.1186/s12882-019-1653-y

**Published:** 2019-12-18

**Authors:** Takeo Ishii, Shizuka Takabe, Yuki Yanagawa, Yuko Ohshima, Yasuhiro Kagawa, Atsuko Shibata, Kunio Oyama

**Affiliations:** 1 Zenjinkai Yokohama Daiichi Hospital, Internal Medicine, 2-5-15 Takashima, Nishi-ku, Yokohama, Kanagawa 220-0011 Japan; 2 Department of Medical Science and Cardiorenal Medicine, Yokohama City University, Graduate School of Medicine, 3-9 Fukuura, Kanazawa-ku, Yokohama, 236-0004 Japan

**Keywords:** Peripheral arterial disease, Skin perfusion pressure, Laser doppler flowmetry

## Abstract

**Background:**

A simpler method for detecting atherosclerosis obliterans is required in the clinical setting. Laser Doppler flowmetry (LDF) is easy to perform and can accurately detect deterioration in skin perfusion. We performed LDF for hemodialysis patients to determine the correlations between blood flow in the lower limbs and peripheral arterial disease (PAD).

**Methods:**

This retrospective study included 128 hemodialysis patients. Patients were categorized into the non-PAD group (*n* = 106) and PAD group (*n* = 22), 14 early stage PAD patients were included in the PAD group. We conducted LDF for the plantar area and dorsal area of the foot and examined skin perfusion pressure (SPP) during dialysis.

**Results:**

SPP-Dorsal Area values were 82.1 ± 22.0 mmHg in the non-PAD, and 59.1 ± 20.3 mmHg in PAD group, respectively (*p* < 0.05). The LDF-Plantar blood flow (Qb) values were 32.7 ± 15.5 mL/min in non-PAD group and 21.5 ± 11.3 mL/min in PAD group (*p <* 0.001). A total of 21 non-PAD patients underwent LDF before and during dialysis. The LDF-Plantar-Qb values were 36.5 ± 17.6 mL/min before dialysis and 29.6 ± 17.7 mL/min after dialysis (*p <* 0.05). We adjusted SPP and LDF for PAD using logistic regression, SPP-Dorsal-Area and LDF-P were significantly correlated with PAD (*p* < 0.05). The receiver-operating characteristic curve analysis indicated cut-off values of 20.0 mL/min for LDF-Plantar-Qb during dialysis.

**Conclusion:**

LDF is a simple technique for sensitive detection of early-stage PAD. This assessment will help physicians identify early-stage PAD, including Fontaine stage II in clinical practice, thereby allowing prompt treatment.

## Background

The incidence of critical limb ischemia (CLI) for the general population is approximately 500–1000 per million per year in European and North American populations. The estimated CLI prevalence for people aged 60–90 years is approximately 1.0% (range, 0.5–1.2%) [1]. Conversely, 6.0% of dialysis patients with peripheral arterial disease (PAD) have undergone amputation (range, less than 2.0% in Japan to 10.0% in the United States) in the DOPPS study [2]. Many mechanisms have been used to detect PAD and lower limb ischemia [3, 4]. Kovacs et al. [5] reported that toe pressure and transcutaneous oximetry (TcPO_2_) with exercise were used to detect PAD in 120 patients. The results indicated that the toe brachial index (TBI) with exercise provides a reliable receiver-operating characteristic (ROC) curve for PAD. Conversely, Jalkanen et al. [6] used the crural index to divide patients into five groups, and those in the crural index group IV had unfavorable survival outcomes. Hardman et al. [7] also classified PAD severity based on atherosclerotic lesions and suggested a potential link between PAD severity and therapeutic treatment.

Skin perfusion pressure (SPP) has been considered a useful tool for detecting PAD severity with TcPO_2_ [8]. The disadvantages of SPP are that it is time-consuming, uncomfortable because of the blood pressure cuff used for patients and the pressure created in the lower extremities, and relatively expensive. Recently, Hijden et al. [9] divided patients into low, middle, and high body mass index groups and performed peripheral arterial tonometry, laser Doppler flowmetry (LDF), and digital thermal monitoring to determine endothelial functions; their data suggested that LDF could be used to predict the prevalence of cardiovascular events and endothelial dysfunction.

For hemodialysis patients with a higher prevalence of atherosclerotic disease and amputation, a large volume of blood is removed during each dialysis session. In the Japanese Society of Dialysis (JSDT) guideline [10], Critical Limb Ischemia (CLI) was defined as Fontaine’s classification stage III or IV. However, it is too late to diagnose PAD with rest pain or ulcer with Fontaine stage III and IV to prevent progression of PAD to severe stage. Therefore, early detection of PAD is required in Fontaine II; however, dialysis patients often have complications with bone and joint disorders, and it is difficult to evaluate intermittent claudication of Fontaine stage II. Now, we tried to detect PAD in early stages, and this was the first time we used mini-laser Doppler blood flowmetry to measure blood flow in the lower limbs of hemodialysis patients. The system comprises a laser Doppler probe and a small handheld monitor with a laptop computer (Additional file 1: Figure S1a). This study aimed to determine the correlation between reduction of blood flow in early stages of PAD. We defined PAD as JSDT guideline plus Fontaine stage II. The information gained could be helpful for rapidly diagnosing lower limb ischemia in dialysis patients.

## Methods

### Patients

This retrospective study involved 128 patients with end-stage renal disease (ESRD) who underwent hemodialysis treatment at Zenjinkai group clinics from April 2017 to May 2018. We performed LDF with the PAD4000® (Kaneka, Osaka, Japan) 30 min after starting hemodialysis. Examination was performed from April 2017 to September 2017 on the 128 patients (Table 1), and 21 patients in non-PAD group (Table 1) performed SPP and LDF recurrently (Additional file 2: Table S1) in May 2018. LDF and SPP analysis were performed within 1 week of each other for each patient. SPP and LDF were performed in the plantar area and in the dorsal area of the foot. The values of SPP and LDF were evaluated per the Fontaine stage. One year after this examination, 21 patients in the non-PAD group performed SPP with LDF again to evaluate blood flow reduction during dialysis. LDF was performed before dialysis and 30 min after start of dialysis on these 21 patients, with comparison of the values before and during dialysis in the paired t-test. All dialysis patients’ symptoms were evaluated according to the Fontaine stage. Prevalence of each Fontaine stage in the PAD group and non-PAD group was evaluated and blood flow was compared with each other. Sixteen healthy volunteers performed SPP and LDF, and the values were compared with those of the dialysis group.

**Table 1:** Patient characteristics

	Non-PAD (*n* = 106)			PAD (*n* = 22)			*p*
Age (year)	59.8	(50.0, 70.0)		71.8	(64.0,81.0)		0.0004
Dialysis Vintage (years)	7.0	(3.0, 14.0)		9.0	(7.0, 11.0)		NS
BMI (kg/m^2^)	23.2	±	4.4	22.1	±	4.5	NS
	Yes	No		Yes	No		
DM (y/n %)	30	76	28.30%	13	9	59.10%	0.0115
Sex (male/female)	19	87	82.10%	4	18	81.80%	NS
SBP (mmHg)	149.3	±	21.3	146.2	±	21.9	NS
DBP (mmHg)	81	±	13.7	71.3	±	13.5	0.0056
Online HDF (y/n)	16	106	15.10%	3	22	13.60%	NS
Total Fluid Removal (kg)	3.3	2.7	3.8	2.6	2.2	3.4	0.0134
KtV	1.4	(1.4, 1.5)		1.4	1.3	1.5	0.893
CGR (%)	104.8	(100.0, 109.6)		86.3	(78.3, 94.3)		0.0002
nPCR (g/kg/day)	0.9	(0.9, 1.0)		0.8	(0.8, 0.9)		0.006
Anticoagulant for dialysis n (%)	Hepaline	Low Molecular	Nafamostat	Hepaline	Low Molecular	Nafamostat	
	77 / 72.6%	22 / 20.8%	6 / 5.7%	11 / 50.0%	7 / 31.8%	6 / 5.7%	
Fontaine grade I n (%)	99		93.40%	8		36.40%	
Fontaine grade II n (%)	7		6.60%	3		13.60%	
Fontaine grade III n (%)	0		0%	5		22.70%	
Fontaine grade IV n (%)	0		0%	6		27.30%	
Hb (g/dL)	11.2	±	0.9	10.8	±	1.2	NS
Ht (%)	34.5	±	2.8	34	±	4.0	NS
Fe (μg/dL)	76.1	±	22.5	61.7	±	22.2	0.0114
Ferritin (ng/mL)	41.5	(20.3, 81.7)		60.3	(28, 106.3)		NS
TSAT (%)	26.8	(21.7, 33.9)		24.3	(16.5, 29.8)		NS
BUN (mg/dL)	68.2	(65.5, 70.9)		58.6	(51.8, 65.4)		0.0111
Cr (g/dL)	12.5	(11.9, 13.0)		9.5	(8.5, 10.5)		< 0.0001
UA (mg/dL)	7.6	(6.6, 8.5)		7.2	(5.6, 7.9)		0.0326
CRP (ng/mL)	0.2	(0.1, 0.4)		0.5	(0.1, 1.2)		0.0104
Na (mEq/L)	139.2	(137.3, 140.5)		137.5	(136.2, 139.2)		NS
K (mEq/L)	4.9	(4.5, 5.2)		4.4	(4.2, 4.8)		0.004
Cl (mEq/L)	101.7	(100,0103.3)		101.7	(100, 104.0)		NS
Pi (mg/dL)	5.6	(5.4, 5.9)		5.1	(4.6, 5.7)		0.1148
Ca (mg/dL)	8.5	(8.1, 9.2)		8.2	(7.9, 8.6)		NS
iPTH (pg/mL)	254.5	(224.4, 284.7)		264.9	(194.2, 335.6)		0.7828
Alb (g/mL)	3.7	(3.6, 3.7)		3.3	(3.1, 3.5)		0.0003
Ret (%0)	16.1	(15, 17.1)		16.8	(13.5, 20.1)		0.6495
TP (g/dL)	7.0	(6.7, 7.2)		6.9	(6.7, 7.3)		NS
Tcho (mg/dL)	164.9	(158.7, 171.2)		137.8	(127.1, 148.4)		< 0.0001
TG (mg/dL)	143.1	(125.0, 161.3)		94.7	(74.3, 115.2)		0.0006
LDL (mg/dL)	88.0	(74.0, 114.5)		68.3	(58.8, 82.3)		0.003
HDL (mg/dL)	47.9	(45.1, 50.7)		45.8	(39.7, 51.9)		0.5126
β2MG (mg/L)	28.1	(26.9, 29.2)		31.1	(28.1, 34.1)		0.061
ESA dose (μg/week)	6.6	(3.0, 10.0)		11.3	(4.2, 17.8)		NS
Vitamin D (μg/week)	0.0	(0.0, 2.5)		0.0	(0.0, 2.5)		NS
Cinacalcet (mg/week)	0.0	(0.0350.0)		87.5	(0.0350.0)		NS
	Yes	No	Yes (%)	Yes	No	Yes (%)	
Ca antagonist (y/n)	62	44	58.50%	5	17	22.70%	0.0041
ACE/ARB (y/n)	30	76	28.30%	5	17	22.70%	NS
Alpha blocker (y/n)	5	101	4.70%	2	20	9.10%	NS
Alpha beta blocker (y/n) + B35	35	71	30.00%	6	16	27.30%	NS
Aspirin (y/n)	21	85	19.80%	9	13	40.90%	0.0506
Ticlopidine hydrochloride (y/n)	3	103	2.80%	0	22	0.00%	NS
Clopidogrel sulfate (y/n)	8	98	7.60%	4	18	18.20%	NS
Cilostazol (y/n)	1	105	0.90%	3	19	13.60%	0.016

Systolic Bp, Alb, Cr, and other covariates were decreased in the PAD group.

*SPP* skin perfusion pressure, *LDF* laser Doppler flowmetry, *BMI* body mass index, *SBP* systolic blood pressure, *DBP* diastolic blood pressure, *Hb* hemoglobin, *HT* hypertension, *TSAT* transferrin saturation, *BUN* blood urea nitrogen, *Cr* creatinine, *UA* uric acid, *CRP* C-reactive protein, *iPTH* intact parathyroid hormone, *Alb* albumin, *TP* total protein, *Tcho* total cholesterole, *TG* triglycerides, *LDL* low-density lipoprotein, *HDL* high-density lipoprotein, *β2MG* beta-2 microglobulin, *KtV* measure of dialysis, *CGR* creatinine generation rate, *nPCR* normalized protein catabolic rate, *ESA* erythropoiesis-stimulating agent, *DM* diabetes mellitus, *ACE* angiotensin-converting enzyme, inhibitor, *ARB* angiotensin receptor blocker, *NS* not significant, *y/n* yes/no.

Data are presented as n(%), mean ± sd, or median (interquartile range)

We obtained written informed consent from participants for this research. All patients and healthy volunteers provided consent for this study (local ethics committee approval: 2017–006263).

### LDF measurements.

The JMS Pocket LDF® (JMS Co., Ltd., Tokyo, Japan) was used to perform measurements. Measurements were obtained using LDF probes that were attached to the skin in the dorsal and plantar areas of the foot (Additional file 1: Figure S1a). Laser beams were then produced by a semiconductor laser diode that was installed in the LDF probes. These beams penetrated the skin and hit the red blood cells in the vasculature, where they dispersed. The laser beams were then converted into scattered light by frequency variation (Doppler shift), which was recognized as electrical signals by a photodetector. The following equation shows the relative value (Q) [10–14]: Q = K ∫ω · P(ω) · dω/I^2^, where K is a constant, Q represents the relative value for blood flow, P(ω) is the special density of the Doppler signal, and I is the scattered light intensity from the tissue (Additional file 1: Figure S1a). The results were expressed as mL/min for blood flow (Qb), mL/min for pulse amplitude (PA), and beats per minute (bpm) for pulse rate. PA was calculated from Qb data and indicated the amplitude of Qb [15]. The Pocket LDF® model consisted of a laser Doppler blood flowmeter (JMS Co., Ltd.).

### SPP measurements

To measure SPP, a PAD4000® (Kaneka, Osaka, Japan) was used according to the method described by Castronuovo et al. [16]. Briefly, SPP was measured with the patient in the supine position in a room maintained at room temperature (approximately 24 °C), and a probe was wrapped around the patient’s foot [3]. SPP was measured according to methods established by previous studies [16–21]. We performed LDF with the PAD4000® (Kaneka, Osaka, Japan) 30 min after starting hemodialysis. LDF and SPP analysis were performed within 1 week of each other for each patient. Blood flow in the plantar area and in the dorsal area of the foot were measured. Secondary, LDF was performed for 21 patients in the non-PAD group 1 year after the first examination. LDF was performed before dialysis and 30 min after the start of dialysis. The LDF values of both groups were compared. All dialysis patients were divided into four groups based on their symptoms and according to the Fontaine classification. The groups were then compared with each other.

Further, SPP analysis and LDF were performed for the healthy control group, and the values were compared to those of the dialysis group.

Each measurement was performed in the dorsal area and in the plantar area, and the values are indicated as an average or median of the average of each right and left results.

### Definition of the Fontaine classification

The classification system used in this study was published in 1954 by Fontaine [22]. This classification system grades the clinical presentation of patients with four distinct stages [7]. All participants were divided according to the Fontaine classification as follows: stage I, asymptomatic and incomplete blood vessel obstruction; stage II, mild claudication and limb pain or claudication at a distance of more than 200 m; stage III, pain during rest, mostly in the feet; stage IV, necrosis and/or gangrene of the limb.

### Diagnosis of PAD

Lower extremity PAD was diagnosed using the obtained claim data for PAD according to the diagnosis criteria. We further defined PAD in dialysis patients who had symptoms of at least intermittent claudication as Fontaine grade II, and further included Fontaine stage III and IV for early detection in dialysis clinics. These data were based on the diagnosis criteria declared by the Japanese Society of Dialysis Therapy (JSDT) [23]. Briefly, the criteria and methods used for diagnosis included the presence of skin ulceration, no palpable lower limb artery, Ratschow’s stress test, ankle-brachial index less than 0.9 [24], multi-detector row computed tomography, and lower limb magnetic resonance imaging [8].

The aim of defining PAD as using Fontaine stage II over/and JSDT criteria is the early detection of PAD for preventive treatment of PAD in dialysis patients.

### Statistical analyses

An unpaired t-test was used to compare the PAD and non-PAD groups to evaluate differences in demographics, laboratory data, and SPP measurements with parametric data. In case of non parametric data, we used Mann-Whitney’s U test to evaluated differences in LDF, laboratory data. To evaluate normal distribution, we fitted Shapiro-Wilk to evaluate parametric or non-parametric data for each covariates.

A paired t-test was used to evaluate examination results before and after dialysis. Significance was defined as *p* < 0.05. Univariate and multivariate logistic analyses of PAD were performed. Multivariate logistic regression with a stepwise method was based on SAS® (SAS, Cary, NY). A ROC analysis to determine the cut-off point was based on this logistic analysis, and the cut-off point was defined according to Youden’s method [25–27]. A further covariate-adjusted ROC analysis was performed based on the logistic analysis using covariates obtained from the stepwise method for logistic analysis of outcomes [27–30]. All statistical analyses were performed using SAS 9.3 (SAS).

## Results

### Patient characteristics

A total of 128 hemodialysis patients were included in this retrospective observational study. Age, Dialysis Vintage, BMI, Systolic BP were recorded. Non-PAD group was 106 cases, and PAD group was 22 cases. Diabetes mellitus (DM) was complicated in 28.3 and 59.1% of the non-PAD and PAD groups, respectively (*p* = 0.0115). Fontaine stages were I, 36.4%; II,13.6%; III,22.7%; IV, 27.3% in the PAD group, but in the non-PAD group, they were stage I, 93.4%; stage II, 6.6%. (Table 1).

### SPP measurements

The average dorsal area SPP (SPP-Dorsal Area) values were 82.1 ± 22.0 and 59.1 ± 20.3 mmHg for the non-PAD and PAD groups, respectively (*p <* 0.0001). The average plantar area SPP (SPP-Plantar Area) values were 80.1 ± 20.2 and 66.0 ± 24.3 mmHg for the non-PAD and PAD groups, respectively (*p =* 0.0161) (Table 2). The SPP-Dorsal- Area values were 88.4 ± 11.8 mmHg for the healthy volunteers group (Additional file 2: Table S2).

**Table 2:** Measurements of the lower limbs of dialysis patients in the non-PAD and PAD groups

SPP mmHg	PAD (−) (*n* = 106)		PAD (+) (*n* = 22)		*p*
	mean	sd	mean	sd	
SPP-Dorsal-Area	82.1	22.0	59.1	20.3	*p* < 0.0001
SPP-Plantar-Area	80.1	20.2	66.0	24.3	*p* = 0.0161
LDF	median	quartile range	median	quartile range	
LDF-Dorsal-Qb	10.7	(8.4, 14.6)	12.0	(8.1, 16.3)	NS
LDF-Plantar-Qb	30.3	(19.7, 43.5)	21.5	(15.0, 26.8)	*p* = 0.0019
LDF-Dorsal-PA	3.1	(2.2, 4.2)	2.2	(1.3, 4.2)	NS
LDF-Plantar-PA	7.9	(5.0, 12.2)	5.2	(2.1, 7.3)	*p* = 0.0031
LDF-Dorsal-PR	77.1	(70.7, 83.3)	79.2	(79.2, 72.9)	NS
LDF-Plantar-PR	74.3	(74.3, 66.6)	78.3	(78.3, 69.9)	NS

Each measurement was performed in the dorsal area and palmar area, and values were indicated as an average of the right and left.

*PAD* peripheral arterial disease, *SPP* skin perfusion pressure, *D* dorsal area of the foot, *P* plantar area of the foot, *LDF* laser Doppler flowmetry, saturation, *Qb* blood flow (mL / min), *PA (mL / min),* pulse amplitude PR (bpm), pulse rate, *NS* not significant, *CI* confidence interval, *SD* standard deviation

Data are presented mean ± sd, or median (interquartile range)

### LDF measurements

**Fig. 1:**
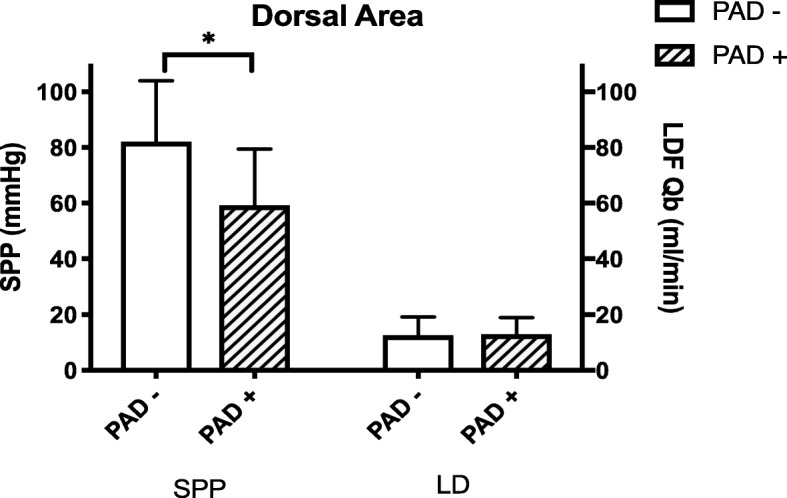
Comparison of the non-PAD and PAD groups using SPP and LDF measurements in the dorsal area. *Statistical significance (*p <* 0.05)

**Fig. 2:**
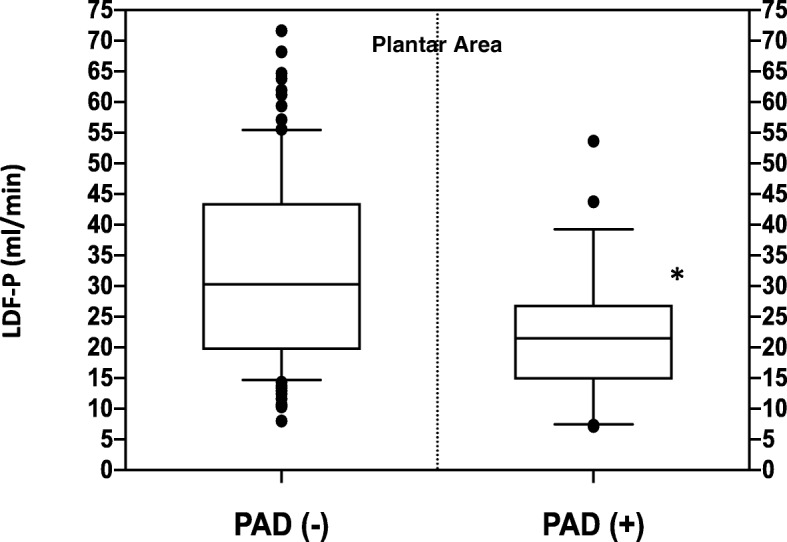
Differences in LDF-Qb for the non-PAD and PAD groups in the plantar area. *Statistical significance (*p <* 0.05)

Blood flow values in the dorsal area (LDF-Dorsal-Qb) were 10.7 (8.5, 14.6) and 12.0 (8.1, 16.3) mL/min in the non-PAD and PAD groups, respectively (not significant) (Fig. 1). Blood flow values in the plantar area (LD-Plantar-Qb) were 30.3 (19.7, 43.5) and 21.5 (15.0, 26.8) mL/min in the non-PAD and PAD groups, respectively (*p* = 0.0019) (Table 2, Fig. 2). The average PA values in the dorsal area (LDF-Dorsal-PA) were 3.1 (2.2, 4.2) and 2.2 (1.3, 4.2) mL/min in the non-PAD and PAD groups, respectively (not significant) (Table 2). However, the LDF-Plantar-PA value in the plantar area was 7.9 (5.0, 12.2) mL/min in the non-PAD group, and it significantly decreased to 5.2 (2.1, 7.3) mL/min in the PAD group (*p* = 0.0031) (Table 2).

### Fontaine classification

**Fig. 3:**
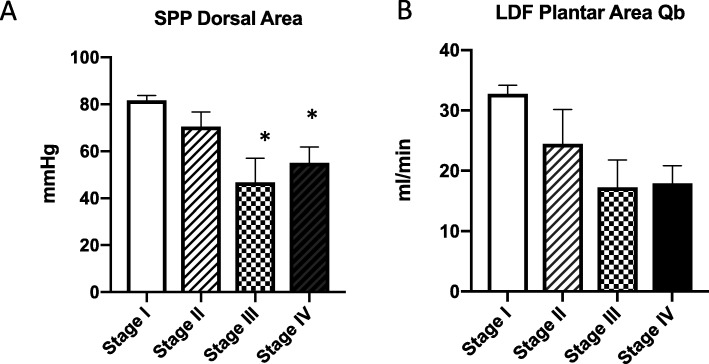
**a** SPP values for the plantar area divided by Fontaine stage. Fontaine stage III and IV were significantly decreased compared with Fontaine grade I in SPP, but stage II was not significant. *Statistical significance (*p <* 0.05). **b** LDF values for the plantar area divided by Fontaine stage. Fontaine stage II was 24.5 +/− 18.0 ml/min decreased from 32.8 +/− 15.0 ml/min

The average SPP-Plantar Area values were 79.2, 79.5, 68.7, and 55.0 mmHg for groups I, II, III, and IV, respectively (Additional file 2: Table S3, Fig. 3a). The LDF-Plantar-Qb values were 32.8, 24.5, 17.3, and 18.0 mL/min for groups I, II, III, and IV, respectively (Additional file 2: Table S3 and Fig. 3b). The proportions of PAD patients with Fontaine grades I, II, III, and IV were 36.4, 13.6, 22.7, and 27.26%, respectively (Table 1).

### Measurements before and during dialysis

One year after the first examination, LDF was performed before dialysis and 30 min after the start of dialysis for 21 patients. The LDF-Plantar-Qb values were 31.0 (22.9, 46.9) and 22.5 (20.1, 32.0) mL/min before and after dialysis, respectively (Additional file 1: Figure S1 g, Additional file 2: Table S1).

### Comparison with healthy volunteers

For the healthy volunteer group, the SPP-Dorsal Area value was 88.4 ± 11.8 mmHg (Additional file 2: Table S2). The LDF-Plantar-Qb value for healthy volunteer group was 19.0 (9.9, 29.0) mL/min (Additional file 2: Table S2, Additional file 1: Figure S1b, S1c).

### Logistic analysis and ROC curves

**Fig. 4:**
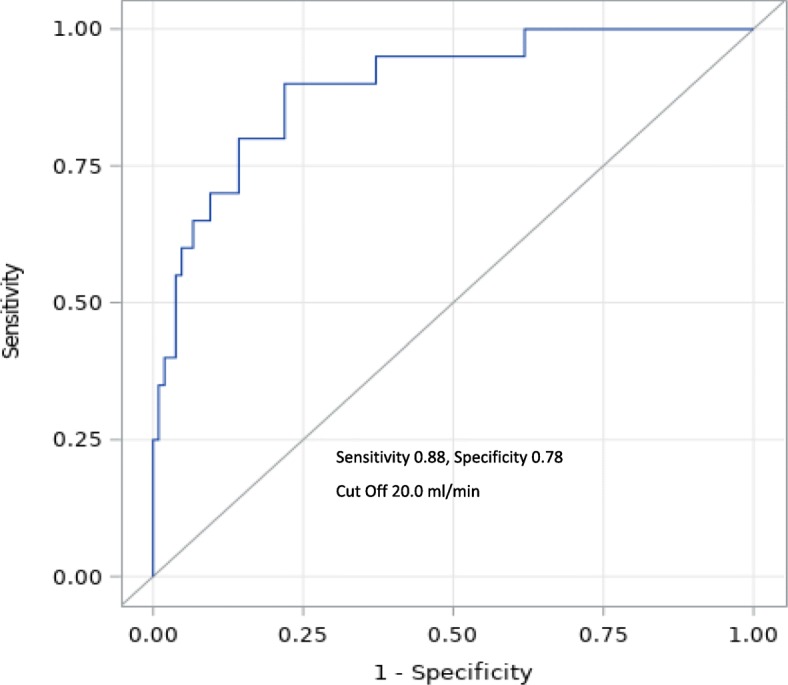
ROC curves of LDF-Plantar-Qb for PAD (adjusted for C-reactive protein, creatinine, total cholesterol). Sensitivity, 0.88; specificity, 0.78; cut-off, 20.0 mL/min using Youden’s index method (Additional file 2: Table S4). PAD(−). without peripheral arterial disease; PAD(+) with peripheral arterial disease; SPP, skin perfusion pressure; LDF, Laser Doppler flowmetry; LDF-Dorsal-Qb, LDF blood flow rate in the dorsal area of the foot; LDF-Plantar-Qb, LDF blood flow rate in the plantar area of the foot; LDF-Qb, LDF blood flow rate; SPP-Dorsal Area, skin perfusion pressure in the dorsal area of the foot; ROC, receiver-operating characteristic

We performed a univariate logistic analysis for all covariates of PAD, which indicated that many of the covariates were correlated with PAD (Additional file 2: Table S5). To stabilize the logistic analysis, covariates with *p* < 0.05 in the univariate logistic analysis were chosen for the multivariate analysis without therapeutic intervention. A stepwise method was performed for the multivariate analysis. SPP and LDF confounded each other; therefore, we performed a divided analysis using a stepwise method. During SPP logistic analysis using the stepwise methods, SPP-Dorsal Area (but not SPP-Plantar Area), creatinine, C-reactive protein, and total choline were chosen as covariates, and all of these covariates were significantly correlated with PAD (*p* < 0.05). The odds ratio for SPP-Dorsal Area was 0.968 (95% confidence interval [CI], 0.94–0.995; *p* = 0.024). A stepwise analysis was conducted that indicated that LDF-Plantar-Qb (but not LDF-Dorsal-Qb), creatinine, C-reactive protein, and total choline were chosen as covariates for the multivariate logistic regression model, and all of these covariates were significantly correlated with PAD (*p* < 0.05). The odds ratio for LDF-Plantar-Qb was 0.935 (95% CI, 0.874–0.985; *p* = 0.026) (Table 3). After logistic regression, a covariate-adjusted ROC analysis was performed using the same covariates detected using the stepwise method (creatinine, C-reactive protein, total choline), 20.0 mL/min for LDF-Plantar-Qb were defined as the cut-off points for PAD (Additional file 2: Table S4) (area under the curve: 0.90 in LD) (Fig. 4).

**Table 3:** Multivariate logistic regression (stepwise method) for PAD using SAS®

Multivariate logistic regression (SPP)				
Covariate	p	Odds ratio	Lower limit	Upper limit
SPP-Dorsal Area	0.0244	0.97	0.94	1.00
Cr	0.0095	0.72	0.56	0.92
CRP	0.0325	2.62	1.18	6.94
Tcho	0.0103	0.97	0.94	0.99
Multivariate logistic regression (laser Doppler)				
Covariate	p	Odds ratio	Lower limit	Upper limit
LDF-Plantar-Qb	0.026	0.94	0.87	0.99
Cr	0.0008	0.65	0.49	0.82
CRP	0.0221	3.08	1.27	8.74
Tcho	0.0176	0.97	0.94	0.99

Skin perfusion pressure (SPP) and laser Doppler (LD) confounded each other; therefore, divided regression was performed.

*PAD* peripheral arterial disease, *D* dorsal area of the foot, *P* plantar area of the foot, *Cr* creatinine, *CRP* C-reactive protein, *Tcho* total choline

We performed sensitivity analysis for cut-off points of the LDF-Plantar-Qb in early-stage PAD. According to the JSDT guideline [23], we included 107 patients with SPP > 50 mmHg as early-stage PAD, and investigated ROC analysis to detect the cut off points for PAD. 14 patients was complicated PAD in this group adjusted with Cr, CRP and Tcho. The result was that LDF-Plantar-Qb 20.1 ml/min was defined as the cut off points (Sensitivity 0.8, Specificity 0.8) (Additional file 2: Table S6).

## Discussion

To our knowledge, this is the first study to evaluate skin microcirculation using LDF for the lower limbs of dialysis patients with or without PAD and to compare the results with those of healthy controls, which allowed for further investigation of skin blood flow changes during dialysis. Early-stage diagnosis of PAD in dialysis patients to prevent deterioration in blood flow, ulcers, necrosis, and systemic infections is critical. Furthermore, surveillance of PAD is required, but the current technology used for SPP measurement only comprises large machines that are not portable, potentially leading to longer time required for the diagnosis. The pocket LDF is handheld and has a small sensor compatible with a laptop computer. Its small size makes the system easily transportable to various clinics for diagnosing PAD.

In conjunction with LDF, the mechanism used to measure the capillary blood flow directly under the skin during dialysis has been previously reported [28–30]. This method was developed to measure the blood flow at the skin surface and to evaluate the function of autonomic neurons. Autonomic dysfunction could be sensitively detected by comparing ear blood flow using LDF for patients with and without DM. In this study, a small and sensitive system was applied to detect PAD among dialysis patients and measure the lower limb blood flow on the skin surface of the dorsal and plantar areas of the foot. According to the logistic regression, SPP-Dorsal Area was significantly correlated with outcomes, but not with SPP-Plantar Area, and LDF-Plantar-Qb was significantly correlated with outcomes, but not significantly correlated with LDF-Dorsal-Qb. We inferred that SPP-Dorsal Area detected blood pressure of the dorsal arteries, whereas the LDF measurement was responsive to microperfusion of the skin. The dorsalis pedis artery is located nearer to the dorsal surface than to the plantar area of the foot. Therefore, SPP more accurately reflects micro-blood pressure in the dorsal area, but the dorsal area is rich with tendons and the bulb of the hair root, which has less micro-blood flow than the plantar area, resulting in a more accurate reflection of skin micro-perfusion than SPP in the plantar area.

Based on the univariate logistic analysis of PAD, covariates with *p* < 0.05 were included in the multivariate analysis (serum creatinine, C-reactive protein, total choline, SPP-Dorsal Area, and LDF-Plantar-Qb) and were detected as the final covariates. We attempted to evaluate the cut-off value for both SPP-Dorsal Area and LDF-Plantar-Qb. With real-world data, we would evaluate the outcomes with the multivariate analysis, but not with the univariate logistic analysis. Nevertheless, using the ROC analysis, outcomes were evaluated based on the univariate logistic analysis. Based on this method, the ROC analysis should be performed with the multivariate logistic analysis [31–34] as covariate-adjusted ROC curves. We adopted the same covariates as those used in the stepwise multivariate logistic analysis (creatinine, C-reactive protein, total choline). For the univariate ROC curves, the cut-off value was 28.5 mL/min (Additional file 1: Figure S1d). However, with adjusted covariates, the cut-off value of LDF-Plantar-Qb was 20.0 mL/min (Fig. 4). The other side adjusted ROC curve of SPP-Dorsal Area was unclear with many similar peaks for outcome (Additional file 1: Figure S1e, f). SPP-Dorsal Area value in Fontaine stage III was significantly decreased to 46.7 +/− 23.2 mmHg (Fig. 3b), but difference between stage I and II was 11.1 mmHg with no significance (Additional file 2: Table S3). The ability to detect PAD among Fontaine stage II might be low in SPP compared with LDF.

SPP measurements were divided according to the Fontaine classification. According to a previous study, the SPP threshold for amputation was 40.0 mmHg [21]. Based on our results, Fontaine group IV had an SPP-Dorsal Area value of 55.1 mmHg. LDF in the plantar area also indicated a similar tendency. Therefore, we suggest that blood flow to the skin in the plantar area of the foot would precisely reflect the blood flow in the lower limb, especially in the peripheral area. Additionally, evaluating the skin blood flow in the plantar area using LDF might provide diagnostic value. If the patient on dialysis had an SPP value of 40.0 mmHg, then it was too late to prevent PAD resection. Fontaine grade II is the first classification involving positive symptoms. Deterioration in blood flow should be predicted during the early stage of PAD and should be diagnosed before the Fontaine classification reaches grade II. LDF-Plantar-Qb indicated a decrease from Fontaine classification grade I. The LDF-Plantar-Qb values were 24.5 ± 18.0 and 17.3 ± 0.1 mL/min for groups II and III, respectively. Therefore, if LDF-Plantar-Qb is less than 20 mL/min, then we should consider initiating therapeutic intervention, including medication (Fig. 4). Impaired endothelial function might be reflected in the PAD group, but this value was consistent for Qb, and PA might be indicated by Qb. Further studies are necessary to assess this association. Overall, there were significant differences in PA between the non-PAD and PAD groups (*p* < 0.0019) (Table 2).

The sensitivity of LDF enables the convenient detection of PAD during an early stage in the clinic, but this sensitivity also causes variations in the measurement results. The LDF measurement values are affected by the temperature on any examination day. It is necessary to adjust the temperature in the room throughout the seasons when LDF is conducted. The temperature in our dialysis room was well-regulated at 24 °C, and most of the study was performed during April and May. Regardless of the season during which the study was conducted, patients waited 40 min to 1 h before the examination in a hospital with a well-regulated temperature system. Therefore, variations in the results because of differences in temperature were limited.

In the healthy volunteer group, the average SPP values were 88.4 ± 11.8 and 96.3 ± 16.6 mmHg in the dorsal and plantar areas, respectively. These values are comparable with those reported by Castronuovo [16]. In the present investigation using healthy controls, SPP-Dorsal Area in the PAD group was relatively higher in the healthy control group than in the non-PAD group (Additional file 1: Figure S1b). In addition, the LDF-Plantar-Qb value of the control group (23.7 ± 20.3 mL/min) was relatively lower than that of the non-PAD group undergoing dialysis (32.7 ± 15.5 mL/min) (Additional file 1: Figure S1c). One reason for this could have been that 53.1% of dialysis patients were treated with calcium antagonists and 27.3% of patients were treated with angiotensin-converting enzyme (ACE) inhibitors/angiotensin receptor blocker (ARB) drugs (Table 1). Debbabi et al. [35] reported that skin blood flow using a laser Doppler device was increased in patients with hypertension who were treated with ACE inhibitors compared with that in the control group and in patients with hypertension who were treated with other drugs. Our results showed that LDF-Plantar-Qb of dialysis patients in the non-PAD group was increased compared with that of the healthy control group; however, it is unknown whether ACE/ARB drug administration affected this result. Another potential reason is that small capillary shunting in the peripheral skin area would be disrupted by aging, uremia, hypertension, diabetes, dyslipidemia, and atherosclerosis. If the terminal arteriole and postcapillary venule shunt at the peripheral skin area are disrupted by these factors, then blood flow would increase compared with that of a younger, healthy patient. Another reason for speculation is that LDF measurements were performed for dialysis patients, and it was suspected that the systemic body fluid increased more than that in the non-dialysis control group. In terms of the fluid volume, it was reported that skin blood flow was affected by hemodialysis [15]. According to the development of fluid removal, LDF blood flow in the plantar area of the foot decreased when systolic blood pressure decreased, suggesting that LDF blood flow is reflective of systemic blood flow or fluid volume.

We performed blood flow LDF before dialysis and after starting dialysis (30 min) for the non-PAD group. Results indicated that the LDF-Planter-Qb significantly decreased after the start of dialysis (Additional file 1: Figure S1 g, Additional file 2: Table S1); however, this did not occur in the dorsal area. This suggested that LDF-Plantar-Qb is mainly affected by the total fluid volume. Together, these factors may affect ACE/ARB, aging, and the uremic state. Furthermore, the value resulting from the second examination using LDF (During Dialysis: LDF-Plantar-Qb 22.5 (20.1, 32.0) mL/min (Additional file 1: Figure S1 g) in the before-and-during study would be close to that in healthy controls (23.7 ± 20.3 mL/min) (Additional file 2: Table S3) according to the first 30 min of fluid removal. Therefore, this deviation requires further investigation.

This sensitive system was also affected by the smoking status of those with DM [35–37]. We compared all dialysis patients with or without DM, including those with lower limb ischemia, and obtained values of 32.6 mL/min and 27.1 mL/min for the non-DM and DM groups, respectively. The prevalence of DM was significantly higher in the PAD group (Table 1) because of the disruption of skin micro-shunting. This result suggested that the Qb value was affected by vasoconstriction and microvascular or endothelial dysfunction, also LDF-Plantar-PA was significantly decreased in PAD (+) group suggested that PA might reflect endothelial dysfunction or atherosclerosis.

This study had several limitations. First, this was an observational study, and the PAD and non-PAD groups were not randomly divided. However, these groups were useful for making immediate judgments regarding the diagnosis. Second, a before-and-during study was designed for repeated LDF measurements 1 year after the first examination. However, the season and temperature were matched. Third, the prescription frequency for calcium antagonists was higher for the PAD group, but there was no evidence of a relationship between the frequency of calcium antagonist administration and skin blood flow. Fourth, the inter-dialysis period was inconsistent, with SPP and LDF examinations performed only after 1 or 2 days. Finally, information regarding comorbidities could only be obtained for DM and ESRD; therefore, other comorbidities were adjusted according to medications.

## Conclusion

This study has provided useful information to assist in the early diagnosis of limb ischemia for ESRD patients and, potentially, individuals with normal kidney functions, thereby leading to effective treatment for PAD to prevent lower limb resection. LDF is a simple method with high sensitivity that can be used to detect early-stage PAD. Skin perfusion of the plantar area of the foot is more sensitive than that of the dorsal area. Furthermore, LDF-Plantar-Qb less than 20.0 mL/min during dialysis is the threshold for determining the diagnosis of PAD patients undergoing hemodialysis.

## Supplementary information

**Additional file 1: Figure S1.** (a). LDF devices. Left; SPP probe is attached on the dorsal area, plantar area. Middle; LDF probe is attached on the dorsal area, plantar area. Right; LDF handheld devices. Result of Qb is indicated on the small window. To obtain result of only the current Qb, there is no need for a computer. (**b).** SPP of the ESRD PAD(−) and PAD(+) groups compared with that of the healthy volunteer group for the dorsal and plantar areas. (**c).** LDF measurements of the PAD(−) and PAD(+) groups compared with that of the healthy volunteer group in the dorsal and plantar areas. (**d)**. ROC curves for LDF-Plantar-Qb. Each curve indicates the following: adjusted for Cr + CRP + Tcho; adjusted for CRP; adjusted for Cr; adjusted for Tcho; and unadjusted. (**e).** ROC curves for SPP-Dorsal Area. Each curve indicates the following: adjusted for Cr + CRP + Tcho; adjusted for CRP; adjusted for Cr; adjusted for Tcho; and unadjusted. (f). ROC curve of SPP-Dorsal Area for PAD (adjusted for C-reactive protein, creatinine, total choline). Sensitivity, 0.90; specificity, 0.96; cut-off, 74.0 mmHg using Youden’s index method (Additional file 2: Table S1). (g). A total of 21 non-PAD patients underwent LDF before and during dialysis. The LDF-P Qb values were 36.5 ± 17.6 mL/min before dialysis and 29.6 ± 17.7 mL/min after dialysis (*p <* 0.05)

**Additional file 2: Table S1.** Comparison between LDF for non-PAD patients (*n* = 21) before dialysis and 30 min after the start of dialysis. **Table S2.** Lower limb blood flow of the healthy volunteer group (*n* = 16) evaluated with SPP and LDF. **Table S3.** Fontaine classification with LDF and SPP values for the dorsal and palmar areas of the lower extremity. **Table S4.** Cut-off value using Youden’s index method. **Table S5.** Univariate logistic analysis for PAD. **Table S6.** Cut-off value using Youden’s index method for sensitivity analysis (SPP > 50 mmHg).

## Data Availability

The datasets used and/or analyzed during the current study are available from the corresponding author on reasonable request.

## Abbreviations

PAD: Peripheral arterial disease
LDF: Laser Doppler flowmetry
SPP: Skin perfusion pressure
DM: Diabetes mellitus
ACE: Angiotensin-converting enzyme
ARB: Angiotensin receptor blocker
