# Supplementary figures and images for: Laser Doppler blood flowmeter as a useful instrument for the early detection of lower extremity peripheral arterial disease in hemodialysis patients: an observational study
Source: BMC Nephrol. 2019 Dec 18;20:470. doi: 10.1186/s12882-019-1653-y (PMC6921472; doi:10.1186/s12882-019-1653-y)

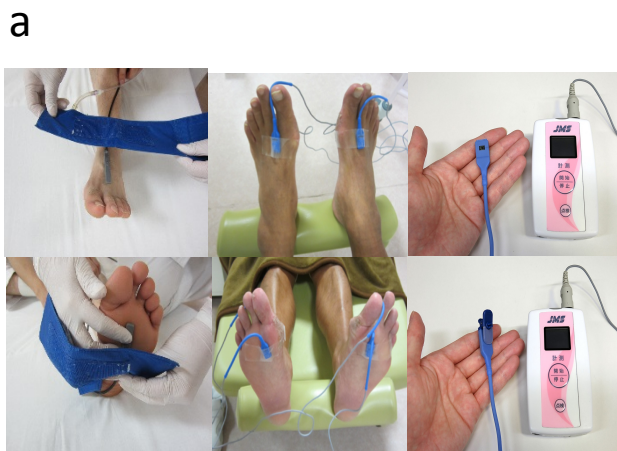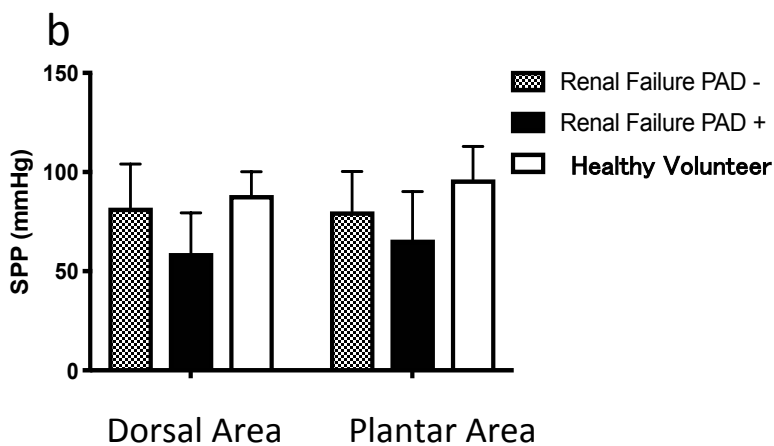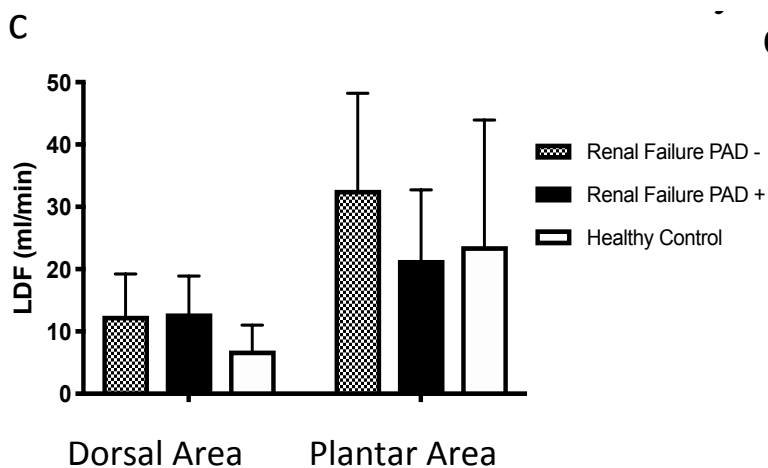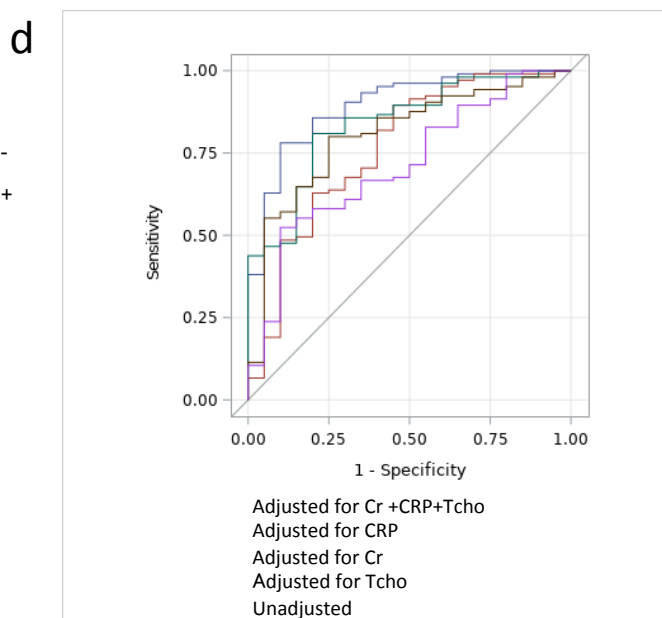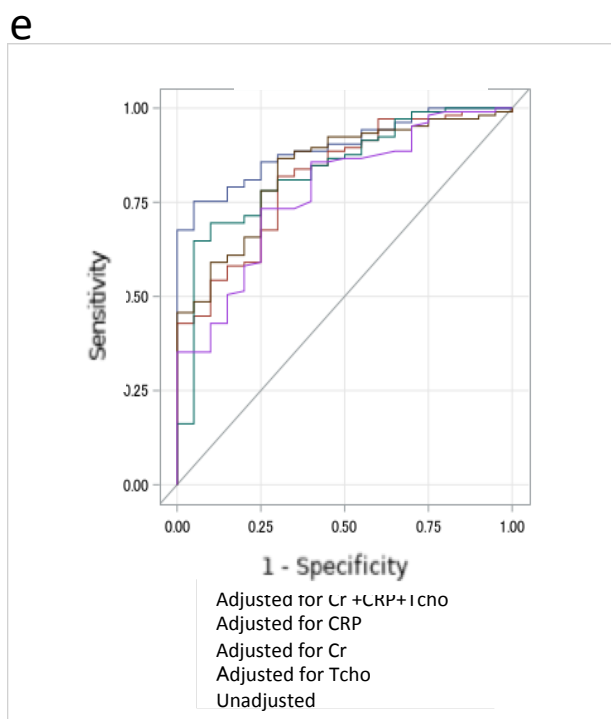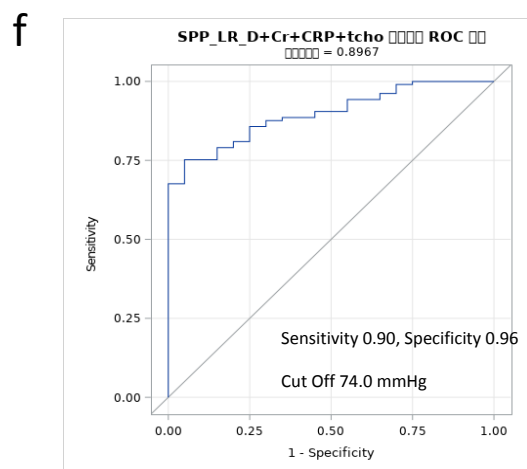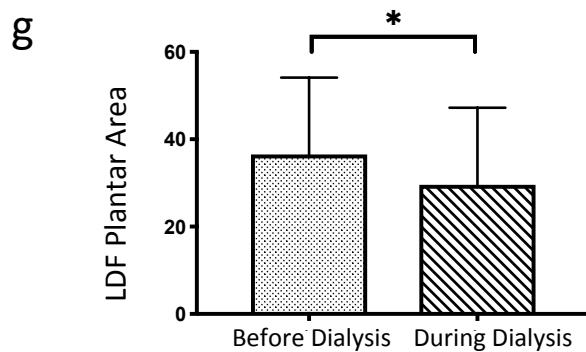

Supplement: Supplementary file 1 — Additional file 1: Figure S1. (a). LDF devices. Left; SPP probe is attached on the dorsal area, plantar area. Middle; LDF probe is attached on the dorsal area, plantar area. Right; LDF handheld devices. Result of Qb is indicated on the small window. To obtain result of only the current Qb, there is no need for a computer. (b). SPP of the ESRD PAD(−) and PAD(+) groups compared with that of the healthy volunteer group for the dorsal and plantar areas. (c). LDF measurements of the PAD(−) and PAD(+) groups compared with that of the healthy volunteer group in the dorsal and plantar areas. (d). ROC curves for LDF-Plantar-Qb. Each curve indicates the following: adjusted for Cr + CRP + Tcho; adjusted for CRP; adjusted for Cr; adjusted for Tcho; and unadjusted. (e). ROC curves for SPP-Dorsal Area. Each curve indicates the following: adjusted for Cr + CRP + Tcho; adjusted for CRP; adjusted for Cr; adjusted for Tcho; and unadjusted. (f). ROC curve of SPP-Dorsal Area for PAD (adjusted for C-reactive protein, creatinine, total choline). Sensitivity, 0.90; specificity, 0.96; cut-off, 74.0 mmHg using Youden’s index method (Additional file 2: Table S1). (g). A total of 21 non-PAD patients underwent LDF before and during dialysis. The LDF-P Qb values were 36.5 ± 17.6 mL/min before dialysis and 29.6 ± 17.7 mL/min after dialysis (p < 0.05) [file 12882_2019_1653_MOESM1_ESM.pdf]
