# Supplementary material for: Laser Doppler blood flowmeter as a useful instrument for the early detection of lower extremity peripheral arterial disease in hemodialysis patients: an observational study
Source: BMC Nephrol. 2019 Dec 18;20:470. doi: 10.1186/s12882-019-1653-y (PMC6921472; doi:10.1186/s12882-019-1653-y)
Supplement: Supplementary file 2 — Additional file 2: Table S1. Comparison between LDF for non-PAD patients (n = 21) before dialysis and 30 min after the start of dialysis. Table S2. Lower limb blood flow of the healthy volunteer group (n = 16) evaluated with SPP and LDF. Table S3. Fontaine classification with LDF and SPP values for the dorsal and palmar areas of the lower extremity. Table S4. Cut-off value using Youden’s index method. Table S5. Univariate logistic analysis for PAD. Table S6. Cut-off value using Youden’s index method for sensitivity analysis (SPP > 50 mmHg). [file 12882_2019_1653_MOESM2_ESM.docx]

Table S1. Comparison between LDF for non-PAD patients (n=21) before dialysis and 30 min after the start of dialysis.

| **n = 21** | **Before Dialysis** | | **During Dialysis** | | **p** |
| --- | --- | --- | --- | --- | --- |
|  | Median | Quartile Range | Median | Quartile Range |  |
| **LDF-Dorsal-Qb (mL/min)** | 12.9 | (9.7 , 17.5) | 15.1 | (10.6 , 19.1) | NS |
| **LDF-Dorsal-PR (bpm)** | 76.5 | (71.2 , 80.3) | 76.4 | (71.3 , 78.8) | NS |
| **LDF-Dorsal-PA (mL/min)** | 3.2 | (2.2 , 5.3) | 3.9 | (2.4 , 5.6) | NS |
| **LDF-Plantar-Qb (mL/min)** | 31.0 | (22.9 , 46.9) | 22.5 | (20.1 , 32.0) | NS |
| **LDF-Plantar-PR (bpm)** | 74.6 | (69.5 , 81.1) | 74.7 | (69.5 , 79.3) | NS |
| **LDF-Plantar-PA (mL/min)** | 7.8 | (5.9 , 14.8) | 7.2 | (5.2 , 11.5) | NS |

| **n = 21** | **Before Dialysis** | | **During Dialysis** | | **p** |
| --- | --- | --- | --- | --- | --- |
|  | Median | Inter Quartile Range | Median | Inter Quartile Range | |
| **LDF-Dorsal-Qb (mL/min)** | 12.9 | (9.7 , 17.5) | 15.1 | (10.6 , 19.1) | NS |
| **LDF-Dorsal-PR (bpm)** | 76.5 | (71.2 , 80.3) | 76.4 | (71.3 , 78.8) | NS |
| **LDF-Dorsal-PA (mL/min)** | 3.2 | (2.2 , 5.3) | 3.9 | (2.4 , 5.6) | NS |
| **LDF-Plantar-Qb (mL/min)** | 31 | (22.9 , 46.9) | 22.5 | (20.1 , 32.0) | NS |
| **LDF-Plantar-PR (bpm)** | 74.6 | (69.5 , 81.1) | 74.7 | (69.5 , 79.3) | NS |
| **LDF-Plantar-PA (mL/min)** | 7.8 | (5.9 , 14.8) | 7.2 | (5.2 , 11.5) | NS |

Postdialysis assessment was performed 30 min after the start of dialysis. LDF-Plantar-Qb was significantly decreased compared to that predialysis (Wilcoxon signed-rank test p<0.05).LDF, Laser Doppler flowmetry; D, dorsal area of the foot; PR, pulse rate; PA, pulse amplitude; Qb, blood flow rate. Datas are presented as n(%), mean ± sd, or median (interquartile range).

Table S2. Lower limb blood flow of the healthy volunteer group (n=16) evaluated with SPP and LDF

| n=16 | mean | sd |
| --- | --- | --- |
| SPP-Dorsal Area (mmHg) | 88.4 | 11.8 |
| SPP-Plantar Area (mmHg) | 96.3 | 16.6 |
|  | median | quartile range |
| LDF-Dorsal-Qb ( ml/min ) | 6.2 | ( 4.4 , 8.0 ) |
| LDF-Dorsal-PR ( bpm ) | 80.0 | ( 78.0 , 84.9 ) |
| LDF-Dorsal-PA ( ml/min ) | 1.8 | ( 1.0 , 2.3 ) |
| LD-Plantar-Qb ( ml/min ) | 19.0 | ( 9.9 , 29.0 ) |
| LD-Plantar-PR ( bpm ) | 78.3 | ( 73.0 , 85.2 ) |
| LD-Plantar-PA ( ml/min ) | 5.0 | ( 3.3 , 9.2 ) |

**Median LDF-Plantar-Qb was 19.0 ( 9.9 , 29.0 ) (mL/min) in the healthy group.**

SPP, skin perfusion pressure; LDF, Laser Doppler flowmetry; LDF-Dorsal-Qb, LDF blood flow rate in dorsal area of the foot; LDF-Plantar-Qb, LDF blood flow rate in plantar area of the foot; LDF-Qb, LDF blood flow rate; SPP-Dorsal Area, skin perfusion pressure in the dorsal area of the foot; ROC, receiver-operating characteristic; LDF-Plantar-PA, LDF pulse amplitude in plantar area of the foot; LDF-Plantar-PR, LDF pulse rate in plantar area of the foot.

Datas are presented as n(%), mean ± sd, or median (interquartile range).

Table S3. Fontaine classification with LDF and SPP values for the dorsal and palmar areas of the lower extremity

| **Fontaine stage** | **n** |  | | | |
| --- | --- | --- | --- | --- | --- |
| **1** | 107 | SPP-Dorsal Area | 81.6 | ± | 22.2 |
|  |  | SPP-Plantar Area | 79.2 | ± | 21 |
|  |  | LD-Dorsal-Qb | 10.8 |  | ( 8.4 , 14.6 ) |
|  |  | LD-Plantar-Qb | 30.2 |  | ( 20.2 , 43.5 ) |
| **2** | 10 | SPP-Dorsal Area | 70.6 | ± | 19.4 |
|  |  | SPP-Plantar Area | 79.5 | ± | 24.2 |
|  |  | LD-Dorsal-Qb | 11.9 |  | ( 8.3 , 16.9 ) |
|  |  | LD-Plantar-Qb | 19.1 |  | ( 13.4 , 22.4 ) |
| **3** | 5 | SPP-Dorsal Area | 46.7 | ± | 23.2 |
|  |  | SPP-Plantar Area | 68.7 | ± | 20.8 |
|  |  | LD-Dorsal-Qb | 14.1 |  | ( 6.3 , 17.8 ) |
|  |  | LD-Plantar-Qb | 17.4 |  | ( 7.3 , 26.8 ) |
| **4** | 6 | SPP-Dorsal Area | 55.1 | ± | 16.4 |
|  |  | SPP-Plantar Area | 55.0 | ± | 16.7 |
|  |  | LD-Dorsal-Qb | 11.4 |  | ( 8.2 , 12.1 ) |
|  |  | LD-Plantar-Qb | 17.8 |  | ( 13.8 , 23.3 ) |

Datas are presented as n(%), mean ± sd, or median (interquartile range).

SPP, skin perfusion pressure; LDF, Laser Doppler flowmetry; LDF-Dorsal-Qb, LDF blood flow rate in dorsal area of the foot; LDF-Plantar-Qb, LDF blood flow rate in plantar area of the foot; LDF-Qb, LDF blood flow rate; SPP-Dorsal Area, skin perfusion pressure in the dorsal area of the foot; ROC, receiver operating characteristic.

Table S4. Cut-off value using Youden’s index method

| SPP | | | | | | |
| --- | --- | --- | --- | --- | --- | --- |
| Unadjusted | SPP-Dorsal Area | AUC | Sensitivity | Specificity | Youden | Distance |
|  | 73.5 | 0.776 | 0.727 | 0.726 | 0.454 | 0.386 |
|  | 73.5 | 0.776 | 0.727 | 0.726 | 0.454 | 0.386 |
|  | 75.5 | 0.776 | 0.773 | 0.679 | 0.452 | 0.393 |
|  | 74 | 0.776 | 0.727 | 0.717 | 0.444 | 0.393 |
|  | 76 | 0.776 | 0.773 | 0.67 | 0.443 | 0.401 |
| **Average** | **74.5** | **0.776** | **0.745** | **0.704** | **0.449** | **0.392** |
| Adjusted | SPP-Dorsal Area | AUC | Sensitivity | Specificity | Youden | Distance |
|  | 59.5 | 0.897 | 0.95 | 0.752 | 0.702 | 0.253 |
|  | 81 | 0.897 | 0.95 | 0.743 | 0.693 | 0.262 |
|  | 76 | 0.897 | 0.95 | 0.733 | 0.683 | 0.271 |
|  | 71.5 | 0.897 | 1 | 0.676 | 0.676 | 0.324 |
|  | 82 | 0.897 | 0.95 | 0.724 | 0.674 | 0.281 |
| **Average** | **74** | **0.897** | **0.96** | **0.726** | **0.686** | **0.278** |
| LDF | | | | | | |
| Unadjusted | LDF-Plantar-Qb | AUC | Sensitivity | Specificity | Youden | Distance |
|  | 28.8 | 0.716 | 0.909 | 0.528 | 0.437 | 0.48 |
|  | 28.9 | 0.716 | 0.909 | 0.519 | 0.428 | 0.49 |
|  | 28 | 0.716 | 0.864 | 0.557 | 0.42 | 0.464 |
|  | 28.9 | 0.716 | 0.909 | 0.509 | 0.419 | 0.499 |
|  | 28.1 | 0.716 | 0.864 | 0.547 | 0.411 | 0.473 |
| **Average** | **28.5** | **0.716** | **0.891** | **0.532** | **0.423** | **0.481** |
| Adjusted | LDF-Plantar-Qb | AUC | Sensitivity | Specificity | Youden | Distance |
|  | 17.7 | 0.896 | 0.9 | 0.781 | 0.681 | 0.241 |
|  | 8 | 0.896 | 0.9 | 0.771 | 0.671 | 0.249 |
|  | 23.6 | 0.896 | 0.9 | 0.762 | 0.662 | 0.258 |
|  | 15.1 | 0.896 | 0.8 | 0.857 | 0.657 | 0.246 |
|  | 35.7 | 0.896 | 0.9 | 0.752 | 0.652 | 0.267 |
| **Average** | **20** | **0.896** | **0.88** | **0.785** | **0.665** | **0.252** |

Youden’s index = sensitivity + specificity - 1. In accordance with Youden’s method, five observations were selected, and the average value was used as the cut-off value. The unadjusted estimate of the cut-off value and adjusted cut-off value with Cr, CRP, and Tcho are indicated. SPP, skin perfusion pressure; Cr, creatinine; CRP, C-reactive protein, Tcho, total cholesterol ; AUC, area under the curve

Table S5. Univariate logistic analysis for PAD

|  | **P** | **Exp (estimate)** | **95% CI lower limit** | **Upper limit** |
| --- | --- | --- | --- | --- |
| **LDF-Plantar-Qb (mL/min)** | 0.0031 | 0.94 | 0.89 | 0.97 |
| **LDF-Dorsal-QB (mL/min)** | 0.8168 | 1.01 | 0.94 | 1.07 |
| **SPP-Dorsal Area (mmHg)** | <.0001 | 0.95 | 0.93 | 0.98 |
| **SPP-Plantar Area (mmHg)** | 0.0064 | 0.97 | 0.95 | 0.99 |
| **Age (year)** | 0.0004 | 1.08 | 1.04 | 1.14 |
| **Sex** | 0.9771 | 0.98 | 0.32 | 3.69 |
| **DM (y/n)** | 0.0074 | 3.66 | 1.43 | 9.74 |
| **BMI (kg/m^2^)** | 0.3474 | 0.95 | 0.83 | 1.06 |
| **Dialysis Vintage (year)** | 0.8294 | 0.99 | 0.93 | 1.06 |
| **Total UF (kg)** | 0.0298 | 0.52 | 0.28 | 0.91 |
| **SBP (mmHg)** | 0.5482 | 0.99 | 0.97 | 1.02 |
| **TP (g/dL)** | 0.1566 | 2.15 | 0.75 | 6.38 |
| **Alb (g/mL)** | 0.0002 | 0.05 | 0.01 | 0.22 |
| **LDL (mg/dL)** | 0.0076 | 0.97 | 0.95 | 0.99 |
| **Hb (g/dL)** | 0.1311 | 0.62 | 0.31 | 1.09 |
| **Fe (μg/dL)** | 0.0107 | 0.97 | 0.94 | 0.99 |
| **Ferr (ng/mL)** | 0.7618 | 1.00 | 0.99 | 1.01 |
| **TSAT (%)** | 0.0523 | 0.95 | 0.89 | 1.00 |
| **Cr (g/dL)** | 0.0002 | 0.70 | 0.57 | 0.83 |
| **BUN (mg/dL)** | 0.007 | 0.95 | 0.92 | 0.99 |
| **UA (mg/dL)** | 0.0185 | 0.66 | 0.46 | 0.92 |
| **CRP (ng/mL)** | 0.0045 | 2.17 | 1.29 | 3.85 |
| **Na (mEq/L)** | 0.1547 | 0.87 | 0.72 | 1.05 |
| **K (mEq/L)** | 0.0192 | 0.38 | 0.16 | 0.84 |
| **Cl (mEq/L)** | 0.5765 | 1.05 | 0.90 | 1.23 |
| **Pi (mg/dL)** | 0.0876 | 0.66 | 0.40 | 1.03 |
| **Ca (mg/dL)** | 0.1496 | 0.60 | 0.29 | 1.18 |
| **iPTH (pg/mL)** | 0.7781 | 1.00 | 1.00 | 1.00 |
| **Ret (%0)** | 0.5842 | 1.02 | 0.94 | 1.11 |
| **Tcho (mg/dL)** | 0.0013 | 0.97 | 0.94 | 0.99 |
| **TG (mg/dL)** | 0.0281 | 0.99 | 0.98 | 1.00 |
| **LDL (mg/dL)** | 0.0076 | 0.97 | 0.95 | 0.99 |
| **HDL (mg/dL)** | 0.5368 | 0.99 | 0.95 | 1.02 |
| **β2MG (mg/L)** | 0.0438 | 1.09 | 1.01 | 1.20 |
| **Aspirin** | 0.0383 | 2.80 | 1.04 | 7.41 |
| **Alpha-blocker/beta-blocker** | 0.5999 | 0.76 | 0.26 | 2.03 |
| **Alpha-blocker** | 0.4199 | 2.02 | 0.28 | 10.12 |
| **ACE/ARB** | 0.5945 | 0.75 | 0.23 | 2.08 |
| **Online (y/n)** | 0.8611 | 0.89 | 0.19 | 3.01 |
| **Cinacalcet, mg/week** | 0.3172 | 1.00 | 1.00 | 1.00 |
| **Vitamin D, week** | 0.8728 | 0.99 | 0.87 | 1.11 |
| **ESA, dose/week** | 0.0276 | 1.07 | 1.01 | 1.13 |
| **nPCR (g/kg/day)** | 0.0125 | 0.02 | <0.001 | 0.40 |
| **CGR (%)** | 0.0034 | 0.97 | 0.95 | 0.99 |
| **KtV** | 0.8954 | 1.14 | 0.17 | 7.90 |
| **Ticlopidine hydrochloride (y/n)** | 0.9835 | <0.001 |  | 4.37 |
| **Clopidogrel sulfate (y/n)** | 0.1314 | 2.72 | 0.67 | 9.66 |
| **Cilostazol (y/n)** | 0.0174 | 16.58 | 2.01 | 345.01 |
| **Aspirin (y/n)** | 0.0016 | 4.68 | 1.81 | 12.61 |
| **Anticoagulant for dialysis** | 0.0372 | 0.07 | 0.00 | 0.80 |

PAD, peripheral arterial disease; SPP, skin perfusion pressure; Qb, blood flow rate; PA, pulse amplitude; PR, pulse rate; BMI, body mass index; SBP, systolic blood pressure; Hb, hemoglobin; TSAT, transferrin saturation; BUN, blood urea nitrogen; Cr, creatinine; UA, uric acid; CRP, C-reactive protein; iPTH, intact parathyroid hormone; Alb, albumin; TP, total protein; Tcho, total cholesterol; ACE/ARB, angiotensin-converting enzyme/angiotensin receptor blockers; DM, diabetes mellitus; ESA, erythropoiesis-stimulating agent; TG, triglycerides; LDL, low-density lipoprotein; HDL, high-density lipoprotein; β2MG, beta-2 microglobulin; KtV, measure of dialysis; CGR, creatinine generation rate; nPCR, normalized protein catabolic rate; y/n, yes/no.

Table S6. Cut-off value using Youden’s index method for sensitivity analysis (SPP > 50 mmHg)

| Adjusted | LDF-Plantar-Qb (mL/min) | AUC | Sensitivity | Specificity | Youden |
| --- | --- | --- | --- | --- | --- |
|  | 17.72 | 0.891 | 0.857 | 0.817 | 0.674 |
|  | 18.892 | 0.891 | 0.857 | 0.806 | 0.664 |
|  | 16.804 | 0.891 | 0.857 | 0.796 | 0.653 |
|  | 22.355 | 0.891 | 0.714 | 0.935 | 0.65 |
|  | 24.686 | 0.891 | 0.786 | 0.86 | 0.646 |
| **Average** | **20.092** | **0.891** | **0.814** | **0.843** | **0.657** |

Sensitivity analysis for cut-off points of the LDF-Plantar-Qb in early-stage PAD. According to the JSDT guideline [10], we included 107 patients with SPP > 50 mmHg as early-stage PAD, and investigated ROC analysis to detect the cut off points for PAD. 14 patients was complicated PAD in this group, and the result was that LDF-Plantar-Qb 20.1 ml/min was defined as the cut off points (Sensitivity 0.8, Specificity 0.8). Youden’s index = sensitivity + specificity - 1. In accordance with Youden’s method, five observations were selected, and the average value was used as the cut-off value. The adjusted cut-off value with Cr, CRP, and Tcho are indicated.
